# New gold(III) complexes TGS 121, 404, and 702 show anti-tumor activity in colitis-induced colorectal cancer: an in vitro and in vivo study

**DOI:** 10.1007/s43440-023-00558-1

**Published:** 2023-12-11

**Authors:** Jakub Włodarczyk, Julia Krajewska, Marcin Talar, Łukasz Szeleszczuk, Agata Gurba, Szymon Lipiec, Przemysław Taciak, Remigiusz Szczepaniak, Izabela Młynarczuk-Biały, Jakub Fichna

**Affiliations:** 1 Department of Biochemistry, Chair of Biochemistry and Chemistry, Faculty of Medicine, Medical University of Łódź, Mazowiecka 5, 92-215 Lodz, Poland; 2 Department of General and Oncological Surgery, Faculty of Medicine, Medical University of Łódź, Pomorska 251, 92-213 Lodz, Poland; 3 Department of Organic and Physical Chemistry, Faculty of Pharmacy, Medical University of Warsaw, Banacha 1, 02-093 Warsaw, Poland; 4 Department of Pharmacodynamics, Faculty of Pharmacy, Medical University of Warsaw, Banacha 1 Str., 02-093 Warsaw, Poland; 5 HESA at the Department for Histology and Embryology, Medical University of Warsaw, Chałubińskiego 5, 02-004 Warsaw, Poland; 6 Inwex Ltd., Solidarności 34, 25-323 Kielce, Poland; 7 Department for Histology and Embryology, Medical University of Warsaw, Chałubińskiego 5, 02-004 Warsaw, Poland

**Keywords:** Colorectal cancer, Gold, Carcinogenesis, Anti-tumor, Colitis-associated colorectal cancer

## Abstract

**Background:**

Chronic inflammation in the course of inflammatory bowel disease may result in colon cancer, or colitis-associated colorectal cancer (CACRC). It is well established that CACRC is associated with oxidative stress and secretion of multiple pro-inflammatory cytokines, e.g. tumor necrosis factor-α. Recently, we proved that the administration of gold(III) complexes resulted in the alleviation of acute colitis in mice. The aim of the current study was to assess the antitumor effect of a novel series of gold(III) complexes: TGS 121, 404, 512, 701, 702, and 703.

**Materials:**

Analyzed gold(III) complexes were screened in the in vitro studies using colorectal cancer and normal colon epithelium cell lines, SW480, HT-29, and CCD 841 CoN, and in vivo, in the CACRC mouse model.

**Results:**

Of all tested complexes, TGS 121, 404, and 702 exhibited the strongest anti-tumor effect in in vitro viability assay of colon cancer cell lines and in in vivo CACRC model, in which these complexes decreased the total number of colonic tumors and macroscopic score. We also evidenced that the mechanism of action was linked to the enzymatic antioxidant system and inflammatory cytokines.

**Conclusions:**

TGS 121, 404, and 702 present anti-tumor potential and are an attractive therapeutic option for colorectal cancer.

**Supplementary Information:**

The online version contains supplementary material available at 10.1007/s43440-023-00558-1.

## Introduction

Colorectal cancer (CRC), the third most frequently diagnosed cancer in the world, is characterized by high morbidity and mortality rates [1]. CRC is associated with environmental factors (e.g. smoking, a diet rich in red meat and poor in fibers, alcohol), heredity (e.g. genetic mutations of *MSH1, MLH1, MSH6, APC,* or *NOD2)* or dysregulation of the immune system [2, 3]. Furthermore, increasing evidence indicates a correlation between chronic inflammation and CRC. Namely, a severe long-term complication of ulcerative colitis (UC) and Crohn’s disease (CD), which are inflammatory bowel diseases (IBD), is associated with an increased risk of CRC and may develop into colitis-associated colorectal cancer (CACRC). The cumulative risk of CACRC induced by UC reaches 18%, while that of CD is 8.3% after 30 years of disease [4]. Previous studies report the relation between CACRC and inflammation, nevertheless, the exact mechanism between these two conditions is still unknown. A significant role in the pathogenesis of CACRC is played by the injured epithelia and triggered immune cells [3]. Moreover, the latest studies suggest that the main factor involved in the inflammatory processes is the nuclear factor-kappa B (NF-κB) pathway activated via pro-inflammatory cytokines interleukin (IL)-1, IL-6, and tumor necrosis factor (TNF-α) and consequently persistent stimulation of NF-κB in the epithelial cells leads to the development of CACRC. Hence, anti-inflammatory agents may be considered an effective solution to prevent CACRC [5].

Gold and its biomedical potential have been discussed in multiple reports. Gold nanoparticles (AuNPs) can be applied in cancer therapy or diagnosis, or as “theranostics” which display both functions [6]. Furthermore, AuNPs may be beneficial in IBD [7, 8]. Gold complexes have also been investigated [9, 10] and found to be potential anticancer drugs. Gold(I) complexes, similarly to gold(III) complexes, have been observed to suppress sulfur-containing and selenium enzymes, including glutathione peroxidase, glutathione-S-transferase, thioredoxin reductase, and cysteine protease, and initiate apoptosis. Marmol et al. [11] evaluated an alkynyl gold(I) complex on the CRC Caco-2 cell line. Authors noticed that this complex initiated necroptosis and showed anticancer activity, which can be considered as a potential treatment in case of apoptosis resistance. Trommenschlager et al. reported that gold(I)-coumarin-caffeine-based complexes exhibited antiproliferative properties in the colon, prostate, and breast cancer cell lines [12]. In addition, two of the investigated complexes displayed anti-inflammatory behavior by inhibiting IL-1β production. Furthermore, a gold(III) porphyrin complex showed cytotoxicity in multiple cancer cell lines and was also reported as an anticancer agent in vivo in nasopharyngeal and hepatocellular carcinoma, colon cancer, melanoma, and neuroblastoma [13, 14]. In animal models, a gold(III) complex with tridentate C-deprotonated C^N^C ligand showed anticancer potential in sarcoma and hepatocellular carcinoma [15].

Despite the beneficial potential of gold and its complexes, they are currently used mainly as drugs for rheumatoid arthritis. Nevertheless, auranofin, a gold(I)-containing compound, owing to its anticancer potential in animal models, is currently tested in clinical trials for ovarian and lung cancer. Furthermore, auranofin showed antibacterial activity against *Clostridium difficile* and multiple enterococcal clinical isolates, which may translate to IBD and CACRC treatment [16].

Recently, we evaluated a series of novel gold(III) complexes as anti-inflammatory therapeutics in in vitro and in vivo models and thoroughly presented the molecular modeling analysis [9, 17, 18]. In this study, we further examined the novel gold(III) complexes: TGS 121, 404, 512, 701, 702, and 703 for their anti-tumor potential in vitro, analyzing the viability of colon cancer cell lines, and in vivo, in the azoxymethane-dextran sulphate sodium (AOM/DSS)-induced mouse model of CACRC.

## Materials and methods

### Synthesis of gold(III) complexes

The analyzed gold(III) complexes (*TGS 121, 404, 502, 701, 702, 703)* were synthesized as described earlier [9, 18].

### Cell culture

The colon cancer cell lines HT-29 and SW480, and normal colon epithelium CCD 841 CoN cells were obtained from the American Type Culture Collection (respectively ATCC HTB-38, CCL-228, and CRL-1790). The cells were cultured in Dulbecco’s modified Eagle’s medium supplemented with 10% FBS 1% l-glutamine and 1% penicillin, streptomycin, and neomycin (Gibco, Life Technologies). The cells grew at 37 °C in a humidified atmosphere and 5% CO_2_, according to the standards procedures.

### Cell viability assay

For cell viability experiments, cells were seeded in triplicate, in a 96-well plate in 200 µL of their respective culture medium. A number of seeded cells per well was CCD 841 CoN-6000; SW480-6000 and HT-29-10 000. Twenty four hours later cells were treated with different concentrations of tested compounds and were incubated for 48 h. Cells treated with the vehicle (medium) were considered as controls. Cells were then incubated with 10 µL of 3-(4,5-dimethylthiazol-2-yl)-2,5-diphenyltetrazoliumbromide (MTT) for two hours after, and 200 µL of dimethyl sulfoxide (DMSO) was added directly into the medium in each well and pipetted up and down several times to dissolve the salt solution with shaking for 20 min. The absorbance was measured at 570 nm. The optical density (OD) values were directly correlated with the number of living cells in the well. The percentage of living cells (%) was calculated according to the following equation: OD of treated cells/ OD of control × 100. The dose–response curves were plotted as the log concentration in µg/mL—versus the percentage of viable cells using Prism 9.0.1 (GraphPad Software Inc., La Jolla, CA, USA). The mean lethal concentration (LC50) was determined at 50% of cell death.

Cell surveillance graphs depict exponential decay curve fits and IC50 = LC50s were evaluated from semilogarithmic transformations of the data. LC50 concentration at which the cell viability remained at 50% after exposure to the tested compound was determined by using a best fit to four-parameter sigmoid log(inhibitor)-response curve. *R*^2^ values as a goodness of fit were also determined and given in the results sections.

### Animals and treatment

Male Balb/C mice obtained from the Animal Facility of the University of Lodz, Poland, were 8 weeks old and weighing 22–24 g. The animals were housed at a constant temperature (22–23 °C) and maintained under a 12‐h light/dark cycle with free access to laboratory chow and tap water. After one week of acclimatization, mice were randomly divided into experimental groups. All procedures were approved by the Local Ethical Committee for Animal Research at the Medical University of Lodz with the following numbers: #4/ŁB85/2018 and #13/ŁB130/2019. All efforts were made to minimize animal suffering and reduce the number of animals used.

Colitis-associated colorectal cancer was induced by a single intraperitoneal administration of azoxymethane (AOM) at the dose of 10 mg/kg as described earlier [19, 20]. One week later, the autoclaved drinking water was replaced by a 1.5% (w/v) dextran sodium sulfate (DSS) solution. After one week, DSS was replaced by the autoclaved water for the next 2 weeks. This cycle was then repeated two more times. The body weight, health, and general behaviour of each mouse were monitored at least three times a week. Untreated control mice received vehicle (0.9% NaCl) instead of AOM and autoclaved water during the experimental period.

The following treatment regime based on our previous experience with animal models and TGS complexes administration [9, 18] was proposed: starting from the end of the third week, TGS 121, 404, and 703 were administered intragastrically at the dose of A—1.68 μg/kg and dose B—16.8 μg/kg in the final volume 100 μL every three days until the end of 14 weeks. Control and AOM/DSS groups received 0.9% NaCl alone (100 μL) administered intragastrically. Mice were euthanized after 14 weeks; the colon from each mouse was excised from the ileocecal junction to the anus, cut, and opened longitudinally. The colon samples were analyzed macroscopically for tumour presence and stage of the disease. Then, the distal part of the colon was divided into two parts—one half for histological analysis and the second half for further molecular studies.

### Macroscopic and microscopic examination

A total macroscopic damage score was calculated for each animal as described previously [19, 20]. The macroscopic scoring was based on five parameters: diarrhea (normal = 0; soft = 1; liquid = 2), presence of bleeding (no = 0; yes = 2), edema (no = 0; mild = 1; intense = 2), erythema (no = 0; mild = 1; intense = 2), and adhesions (no adhesion = 0; troublesome dissection = 1; visible adhesions = 2). Further, macroscopic tumors were counted, and the number of tumors (diameter: < 3 mm and > 3 mm) was calculated. To reduce observer bias, the whole macroscopic evaluation was designed in a blinded setup.

For microscopic evaluation, the colonic tissue was fixed in 10% formalin overnight, then routinely dehydrated and embedded in paraffin. Three 5 μm sections per colon were cut and stained with hematoxylin and eosin. Subsequently, photographs were taken using an Axio Imager A2 microscope (Carl Zeiss, Oberkochen, Germany) and a digital imaging system consisting of a digital camera (Axiocam 506 clolor, Carl Zeiss, Germany) and image analysis software (Zen 2.5 blue edition, Carl Zeiss, Germany) with 20 × magnification.

### Determination of tissue myeloperoxidase activity

To monitor the degree of inflammation, myeloperoxidase (MPO) activity was determined using a standardized method. Colon segments (approx. 30 mg) were homogenized in hexadecyltrimethylammonium bromide (HTAB) buffer (0.5% HTAB in 50 mM potassium phosphate buffer, pH 6.0; 50 mg tissue/mL). Homogenates were centrifuged (15 min, 13,200 × g, 4 °C). On a 96-well plate, 200 μL of 50 mM potassium phosphate buffer (pH 6.0), supplemented with 0.167 mg/mL of O-dianisidine hydrochloride and 0.05 μL of 1% hydrogen peroxide, was added to 7 μL of the supernatant. Absorbance was measured at 450 nm after 30 and 60 s (iMARK Microplate Reader, Biorad, Hertfordshire, UK). All measurements were performed in triplicate. MPO activity was expressed in milliunits per gram of wet tissue, 1 unit being the quantity of enzyme able to convert 1 μmol hydrogen peroxide to water in 1 min at room temperature.

### Real-time RT PCR and ELISA assays

The level of the oxidative stress biomarker, heme oxygenase-1 (HO-1), was determined by the HO-1 ELISA Kit (EIAab kit #E0584m, Wuhan, China). Tissue specimens were collected from the distal part of the mice colon affected by tumorigenesis or the corresponding part in the case of control mice and homogenized according to the manufacturer’s instructions. Assay was performed following the provided user’s manual and each sample was assayed in duplicate.

Total RNA was extracted from snap-frozen colonic tissues obtained from experimental and control groups using the Total RNA Mini Plus kit (#036-100, A&A Biotechnology, Gdansk, Poland) according to the manufacturer’s instructions. Following, cDNA was synthesized using a High-Capacity cDNA Reverse Transcriptase Kit (#4368814, Applied Biosystems). Quantitative real-time PCR analysis was performed using the TaqMan probes: Il-1β: Mm00434228_m1; Tnf-α:Mm00443258_m1; transforming growth factor-β (TGF-β):Mm01178820_m1 and TaqMan Gene Expression Master Mix without UNG (#4440040, Applied Biosystems) by manufacturer’s protocol on Lightcycler 96 apparatus (Roche, Warsaw, Poland). The Ct (cycle threshold) values for all tested genes were normalized to Ct values obtained for GAPDH. The relative amount of mRNA copies was calculated using the following equation: 2^[−(Ct tested gene−Ct GAPDH)]^ × 1000.

### Statistics

Results were analyzed in Prism 9 (GraphPad Software Inc., La Jolla, CA, USA) with the use of one-way ANOVA followed by the Tukey post hoc test (or nonparametric the Kruskal–Wallis test followed by the Dunn’s post hoc test, depending on the distribution of variables). When suitable the one-way repeated measures ANOVA was used. The survival rate was evaluated by log-rank test using the Kaplan–Meier method. The data are presented as mean ± standard error of the mean (SEM) or median with 25th and 75th percentiles with minimum and maximum value. *P* values < 0.05 were considered statistically significant.

## Results

### Cell viability

**Fig. 1:**
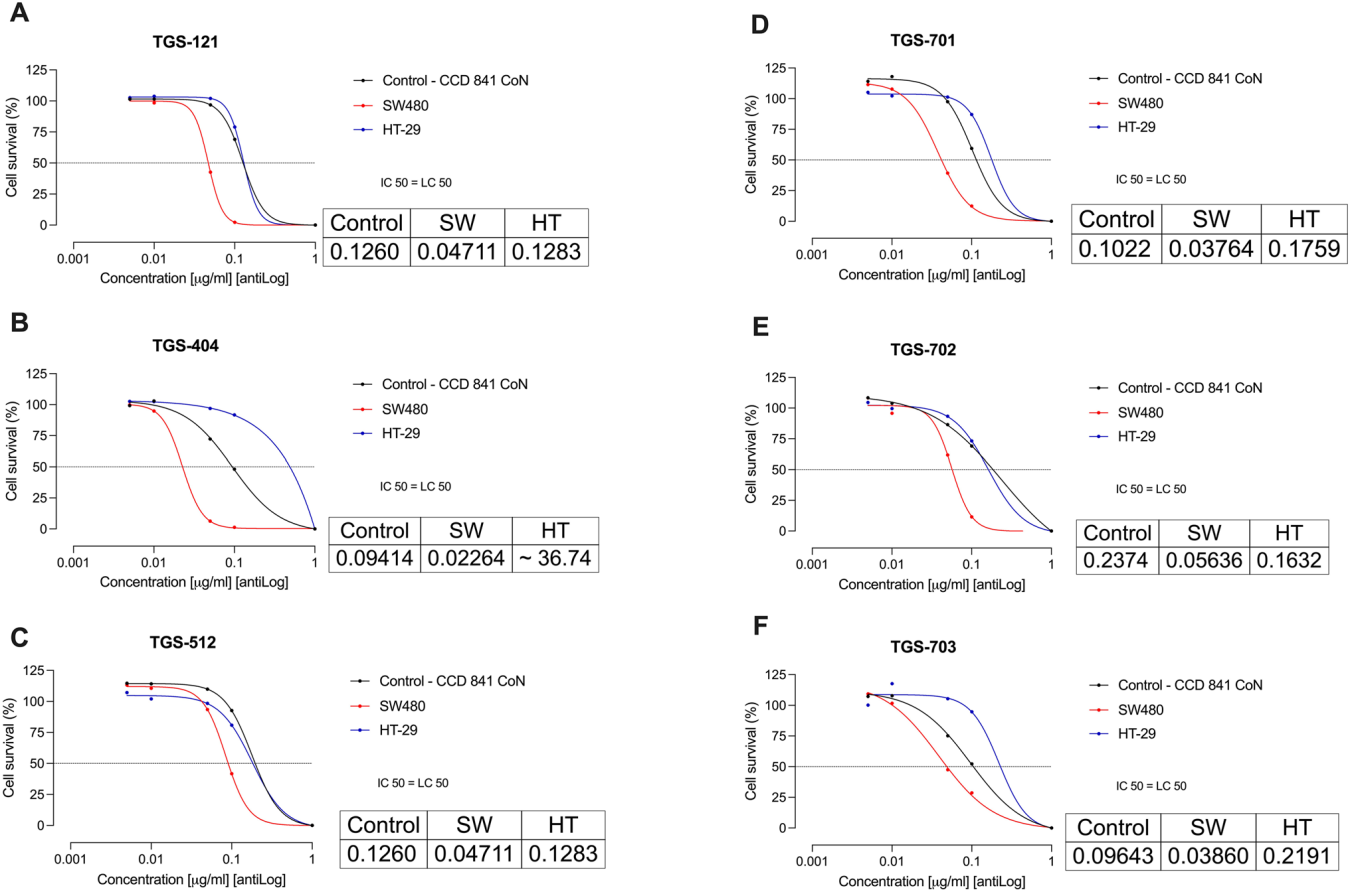
**A**–**F** Dose–response curves representing viability of culture cell lines after treatment with different concentrations of tested TGS complexes. The inserted tables show calculated 50% lethal concentration values (as µg/mL). Data are mean ± SEM

Dose–response curves representing the cell viability percentage (%) of healthy colon epithelial cells (control – CCD 841 CoN) and colon cancer cells (SW480 and HT-29) treated with different concentrations of TGS complexes (0.005–1 ug/ml) are presented in Fig. 1. For all tested TGS complexes, the concentrations needed for 50% growth inhibition were lower for colon cancer cell line SW480 than for healthy colon epithelial cells CCD 841 CoN. HT-29 cells were more resilient and respective LC50 values were greater than in CCD 841 CoN cells in all tested complexes, except for TGS-702.

### TGS complexes exhibited an anti-tumor effect in the AOM/DSS mouse model of CACRC

The CACRC mouse model was established with AOM injection, followed by three cycles of proinflammatory DSS oral administration. In this model, carcinogenesis and tumor development in the distal colon were reported previously [19, 20].

**Fig. 2:**
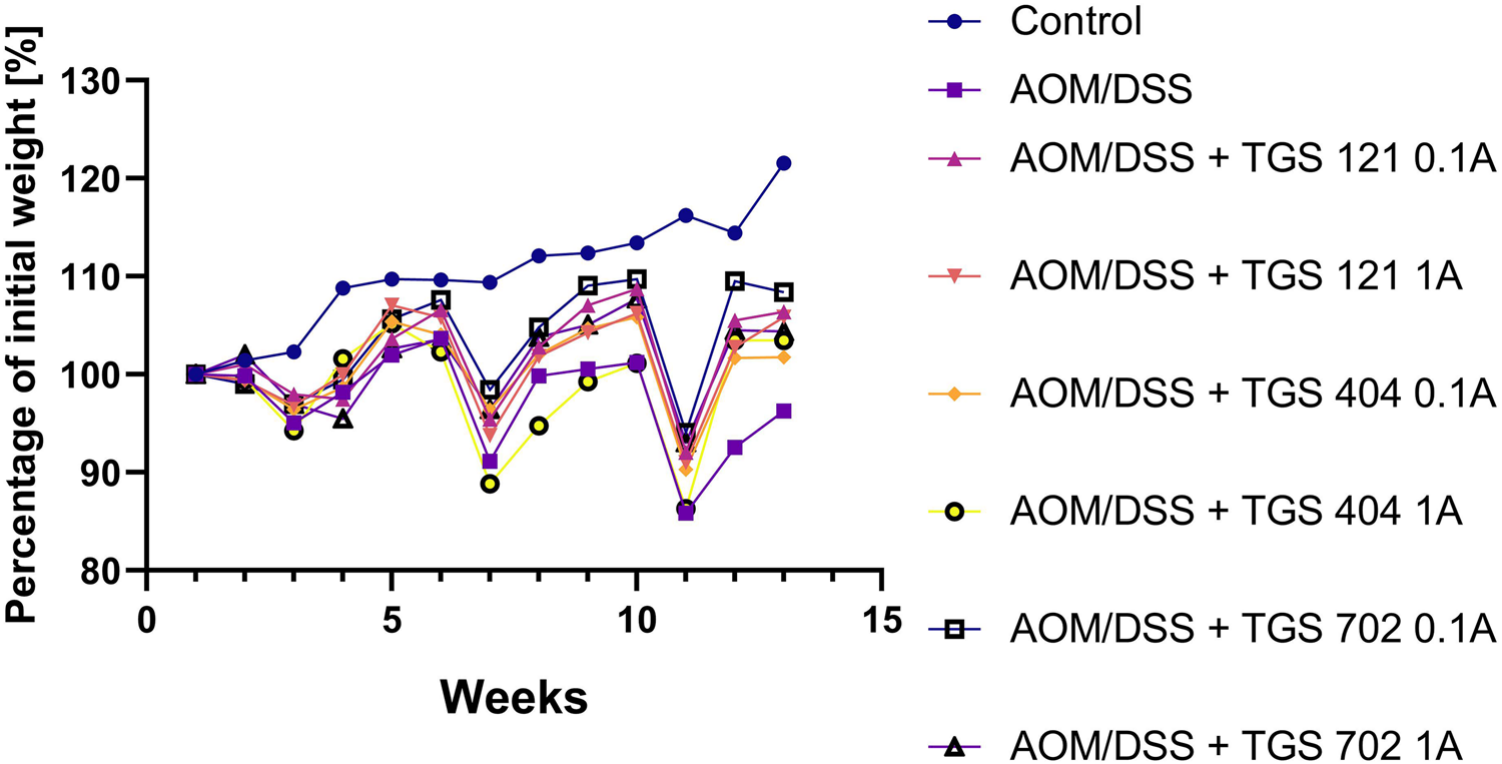
Changes in the weight of mice in the azoxymethane (AOM)/dextran sodium sulfate (DSS)-induced animal model of colitis-associated colorectal cancer. Data are presented as mean ± SEM (*n* = 6–10). Data were analyzed using a one-way repeated measures ANOVA with Geisser-Greenhouse correction followed by Tukey’s post hoc test

**Fig. 3:**
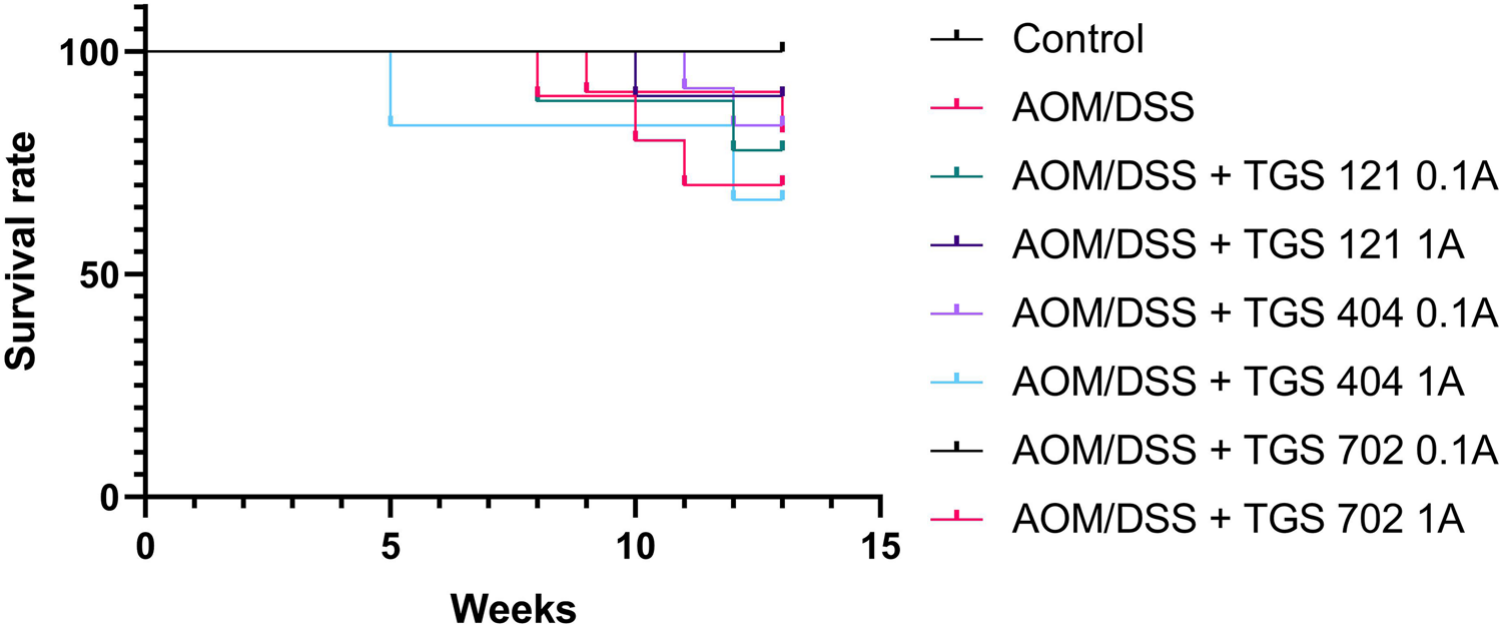
The survival rates of mice in the azoxymethane (AOM)/dextran sodium sulfate (DSS)-induced animal model of colitis-associated colorectal cancer. The survival rate was evaluated by log-rank test using the Kaplan–Meier method

As shown in Fig. 2 and Table 1, administration of TGS complexes to the AOM/DSS mice caused significantly lower weight reduction in every group except TGS 404 1A, as assessed by one-way repeated measures ANOVA with Geisser-Greenhouse correction (F _1.949, 23.39_ = 18.73, *p* < 0.001), followed by Tukey’s post hoc test. However, the AOM/DSS group had a trend of decreased body weight compared with the AOM/DSS + TGS groups. Analysis of the survival rate during the experiment revealed no significant differences between groups (Fig. 3).

**Table 1:** *p*-values from the post-hoc analysis of the changes in the weight of mice in the azoxymethane (AOM)/dextran sodium sulfate (DSS)-induced animal model of colitis-associated colorectal cancer

	Control	AOM/DSS	AOM/DSS + TGS 121 0.1A	AOM/DSS + TGS 121 1A	AOM/DSS + TGS 404 0.1A	AOM/DSS + TGS 404 1A	AOM/DSS + TGS 702 0.1A	AOM/DSS + TGS 702 1A
Control	–	**0.005**	**0.01**	**0.009**	**0.007**	**0.005**	**0.02**	**0.006**
AOM/DSS	**0.005**	–	**0.02**	**0.02**	**0.02**	0.98	**0.02**	0.07
AOM/DSS + TGS 121 0.1A	**0.01**	**0.02**	–	0.8	0.25	0.09	0.11	0.62
AOM/DSS + TGS 121 1A	**0.009**	**0.02**	0.8	–	0.81	0.05	0.11	0.99
AOM/DSS + TGS 404 0.1A	**0.007**	**0.02**	0.25	0.81	–	0.46	**0.03**	0.9
AOM/DSS + TGS 404 1A	**0.005**	0.98	0.09	0.05	0.46	–	**0.03**	0.37
AOM/DSS + TGS 702 0.1A	**0.02**	**0.02**	0.11	0.11	**0.03**	**0.03**	–	0.08
AOM/DSS + TGS 702 1A	**0.006**	0.07	0.62	0.99	0.9	0.37	0.08	–

Bolded values represent statistical significance (*p* < 0.05). Data were analyzed using a one-way repeated measures ANOVA with Geisser-Greenhouse correction followed by Tukey’s post hoc test

**Fig. 4:**
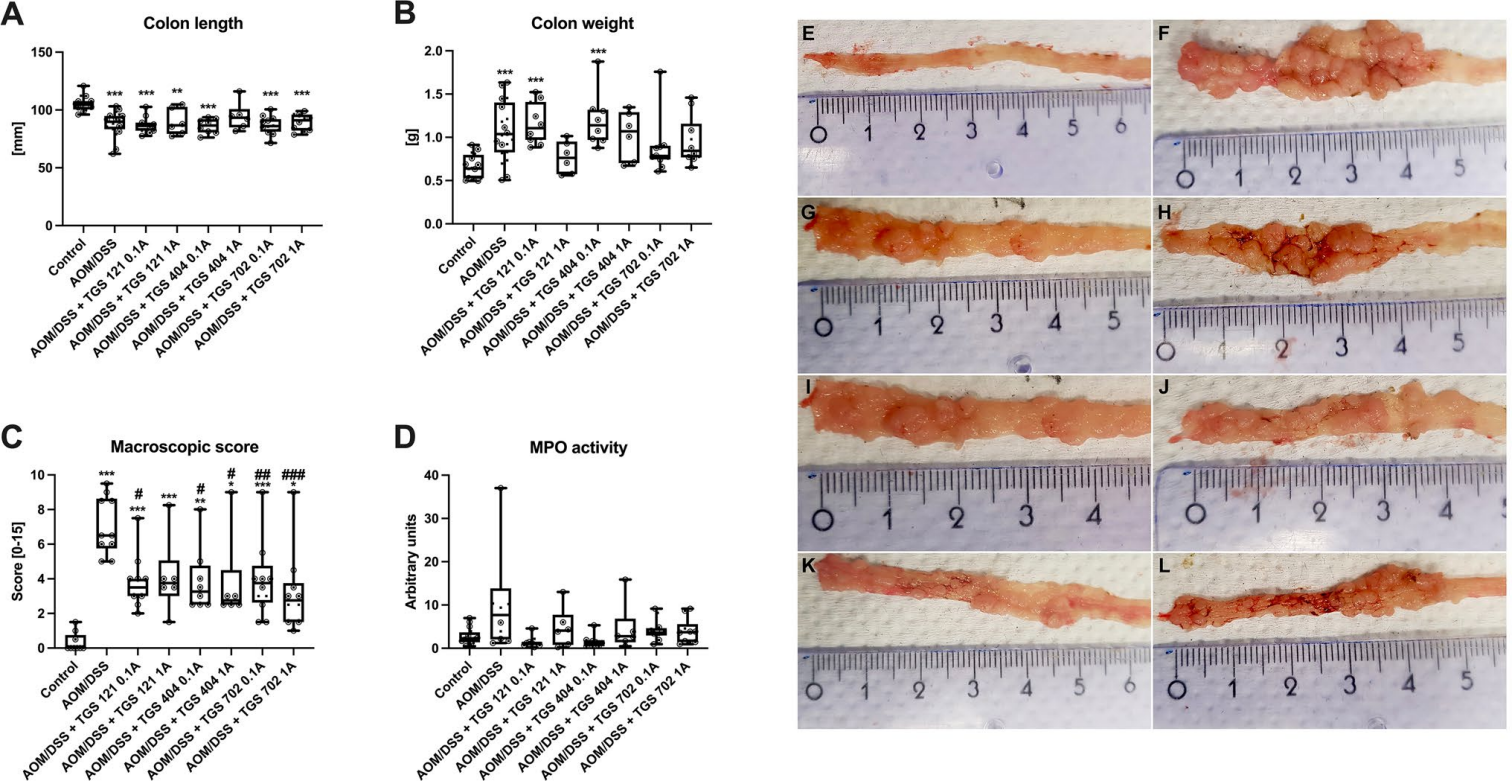
TGS complexes exerted anti-tumor activity in the mouse model of azoxymethane (AOM)/dextran sodium sulfate (DSS)-induced colitis-associated colorectal cancer after 14 weeks, as indicated by the following parameters: colon length (**A**), colon weight (**B**), macroscopic score (**C**), and myeloperoxidase activity (MPO) (**D**). The macroscopic scoring involved five parameters: diarrhea, presence of bleeding, edema, erythema, and adhesions. Data are presented as median with 25th and 75th percentiles with minimum and maximum values (*n* = 6–10). *p < 0.05, **p < 0.01 and ***p < 0.001 as compared with the control mice; ^#^*p* < 0.05, ^##^*p* < 0.01, ^###^*p* < 0.001 as compared with AOM/DSS only animals. Data were analyzed using a one-way ANOVA followed by Tukey’s post hoc test (4A and 4B), and the Kruskal–Wallis test followed by Dunn’s post-hoc test (4C and 4D). Representative images of macroscopic colon samples: control group (**E**), AOM/DSS only group (**F**), AOM/DSS + TGS 121 0.1A (**G**), AOM/DSS + TGS 121 1A (**H**), AOM/DSS + TGS 404 0.1A (**I**), AOM/DSS + TGS 404 1A (**J**), AOM/DSS + TGS 702 0.1A (**K**), AOM/DSS + TGS 702 1A (**L**)

To further evaluate the anti-tumor properties of TGS complexes, we performed a macroscopic examination of the colon. In mice that received AOM/DSS, significant shortening of colon length was observed (F_7, 57_ = 7.974; *p* < 0.001), as assessed by one-way ANOVA followed by post-hoc Tukey test (Fig. 4A). Only mice treated with TGS 404 1A were characterized by similar colon length to the control group, while administration of other tested compounds did not reverse the AOM/DSS-induced shortening of the colon. The colon weight in the AOM/DSS-only group was significantly increased compared to the control group (F_7, 57_ = 5.837; *p* < 0.001), as assessed by one-way ANOVA followed by post-hoc Tukey test (Fig. 4B). Similarly, in AOM/DSS-TGS treated groups the weight was also increased compared to the control group, but only in TGS 121 0.1A and TGS 404 0.1A the statistical significance was observed. Every group stimulated with AOM/DSS developed visible signs of tumorigenesis, which was assessed with a macroscopic score (Fig. 4C) and the treatment with TGS complexes significantly reversed the effect of AOM/DSS alone with the following statistical significance: TGS 121 0.1A-*p* = 0.01, TGS 404 0.1A-*p* = 0.01, TGS 404 1A-*p* = 0.05, TGS 702 0.1A-*p* = 0.009 and TGS 702 1A = p < 0.001, as assessed by the Kruskal–Wallis test followed by post-hoc Dunn’s test (H = 37.81; *p* < 0.001). The assessment of MPO activity, which represents neutrophil infiltration of the intestine and inflammation of tissue revealed no significant differences between groups, as assessed by the Kruskal–Wallis test (H = 16.87; *p* = 0.07) (Fig. 4D). However, a trend, where MPO activity was highest in the AOM/DSS-only group and slightly lower in treated and control groups, was observed. Figure 4E–L shows representative macroscopic images.

**Fig. 5:**
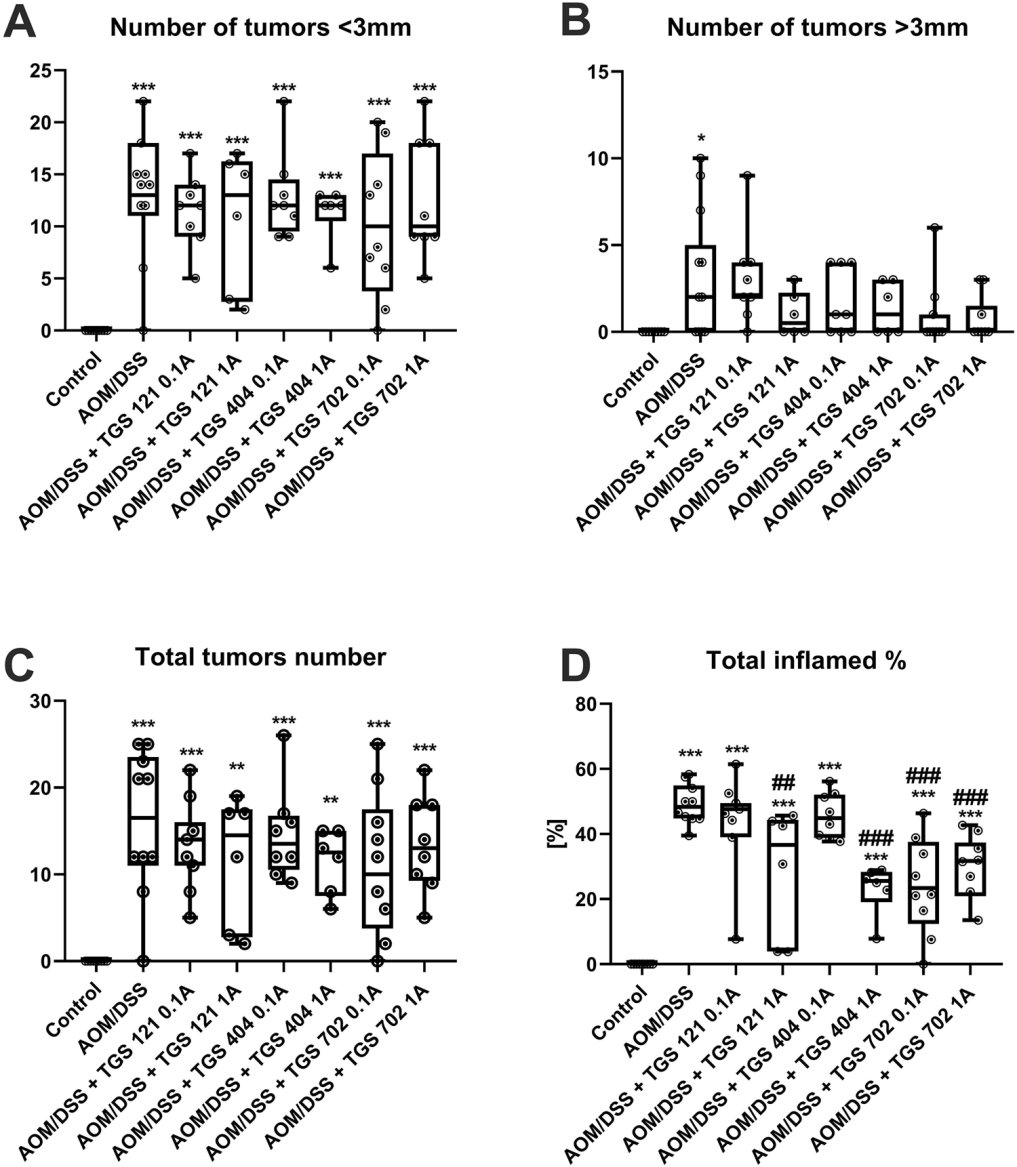
The effect of TGS complexes on tumorigenesis in the azoxymethane (AOM)/dextran sodium sulfate (DSS)-induced model of colitis-associated colorectal cancer (CACRC): number of tumors lower than 3 mm in diameter (**A**), number of tumors greater than 3 mm in diameter (**B**), the total number of tumors (**C**), and percentage of inflamed colonic tissue (**D**) for control, AOM/DSS-only treated mice, and mice with AOM/DSS induced CACRC treated with TGS 121, 404 and 702 in two doses: 1.68 μg/kg (0.1A) and 16.8 μg/kg (1A). Tumors were counted and measured with the use of a caliper with 0.02 mm resolution. Data are presented as median with 25th and 75th percentiles with minimum and maximum values (*n* = 6–10). *p < 0.05, **p < 0.01, and ***p < 0.001 as compared with the control mice; ^##^*p* < 0.01, ^###^*p* < 0.001 as compared with AOM/DSS-only treated animals. Data were analyzed using a one-way ANOVA followed by Tukey’s post hoc test

**Fig. 6:**
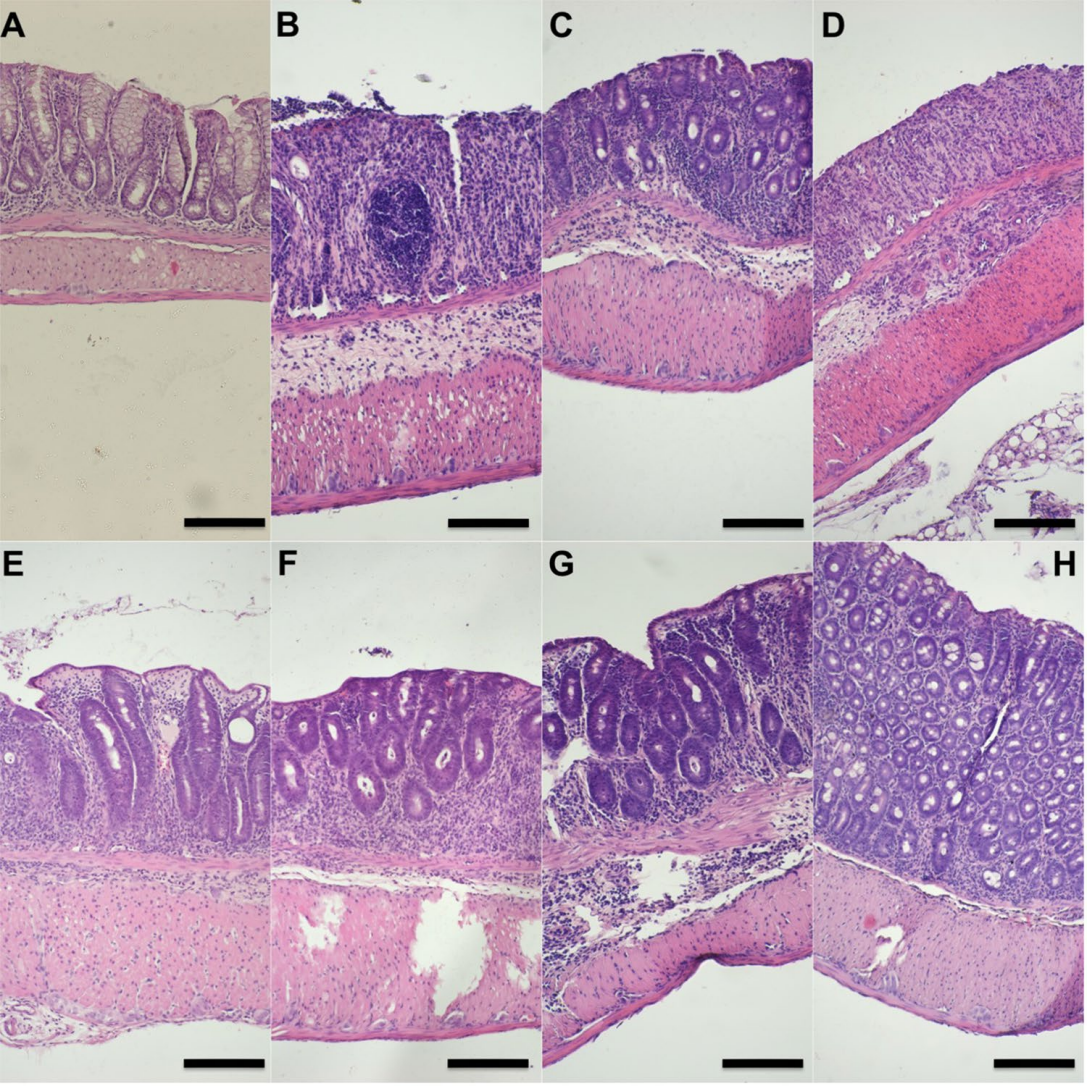
Representative photographs of hematoxylin and eosin staining of colon samples: control group (**A**), azoxymethane (AOM)/dextran sodium sulfate (DSS) only group (**B**), AOM/DSS + TGS 121 0.1A (**C**), AOM/DSS + TGS 121 1A (**D**), AOM/DSS + TGS 404 0.1A (**E**), AOM/DSS + TGS 404 1A (**F**), AOM/DSS + TGS 702 0.1A (**G**), AOM/DSS + TGS 702 1A (**H**). Scale bar = 100 μm

After 14 weeks of experiment tumors were noted in all mice exposed to AOM/DSS; in the control group, no tumors were observed (Fig. 5A–D). The AOM/DSS-only group was characterized by the highest tumor number and greatest tumor area. The lower number of tumors was observed in all groups treated with TGS complexes, however, no statistical significance was stated, as assessed by one-way ANOVA (F_7, 57_ = 11.11; *p* < 0.001) followed by Tukey post-hoc test. In terms of tumor area, administration of TGS 121 1A, TGS 404 1A, TGS 702 0.1A, and 1A significantly diminished the area of the colon covered with tumors compared with the AOM/DSS-only group, as assessed by one-way ANOVA (F_7, 57_ = 15.69; *p* < 0.001) followed by Tukey post-hoc test (Fig. 5D). Figure 6 shows representative microscopic images of pathomorphological changes in colon tissues, which were consistent with clinical and macroscopic changes.

**Fig. 7:**
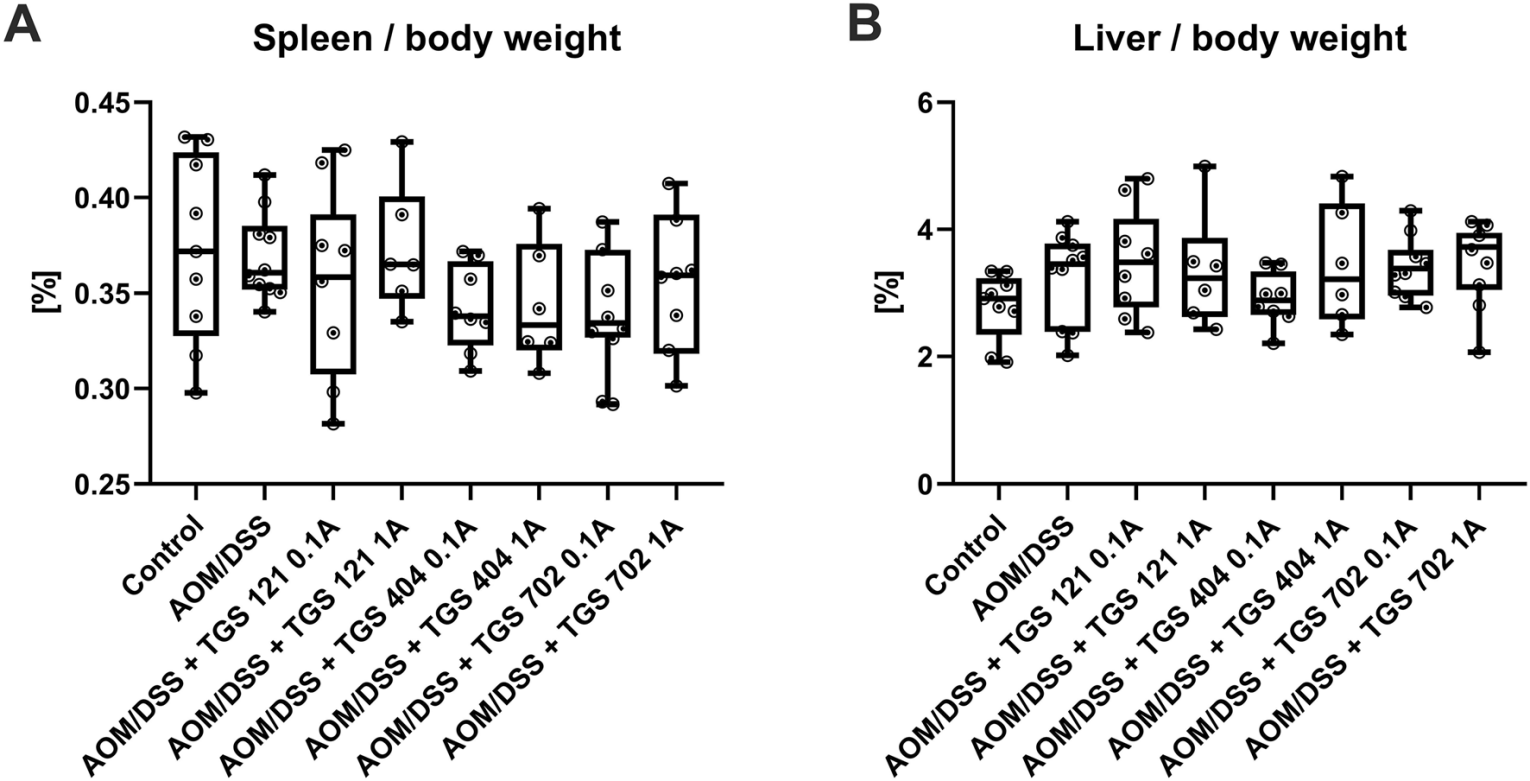
Resected spleen weight to mouse weight ratio (**A**) and resected liver weight to mouse weight ratio (**B**) in the azoxymethane (AOM)/dextran sodium sulfate (DSS)-induced animal model of colitis-associated colorectal cancer. Data are presented as median with 25th and 75th percentiles with minimum and maximum values (*n* = 6–10). Data were analyzed using a one-way ANOVA

As shown in Fig. 7, neither AOM/DSS-only nor TGS complexes administration had any statistically significant effect on the ratio of spleen weight or liver weight to mouse body weight on the scoring day, as assessed by one-way ANOVA (respectively, F_7, 57_ = 2.162, *p* = 0.06 and F_7, 57_ = 1.305, *p* = 0.37).

**Fig. 8:**
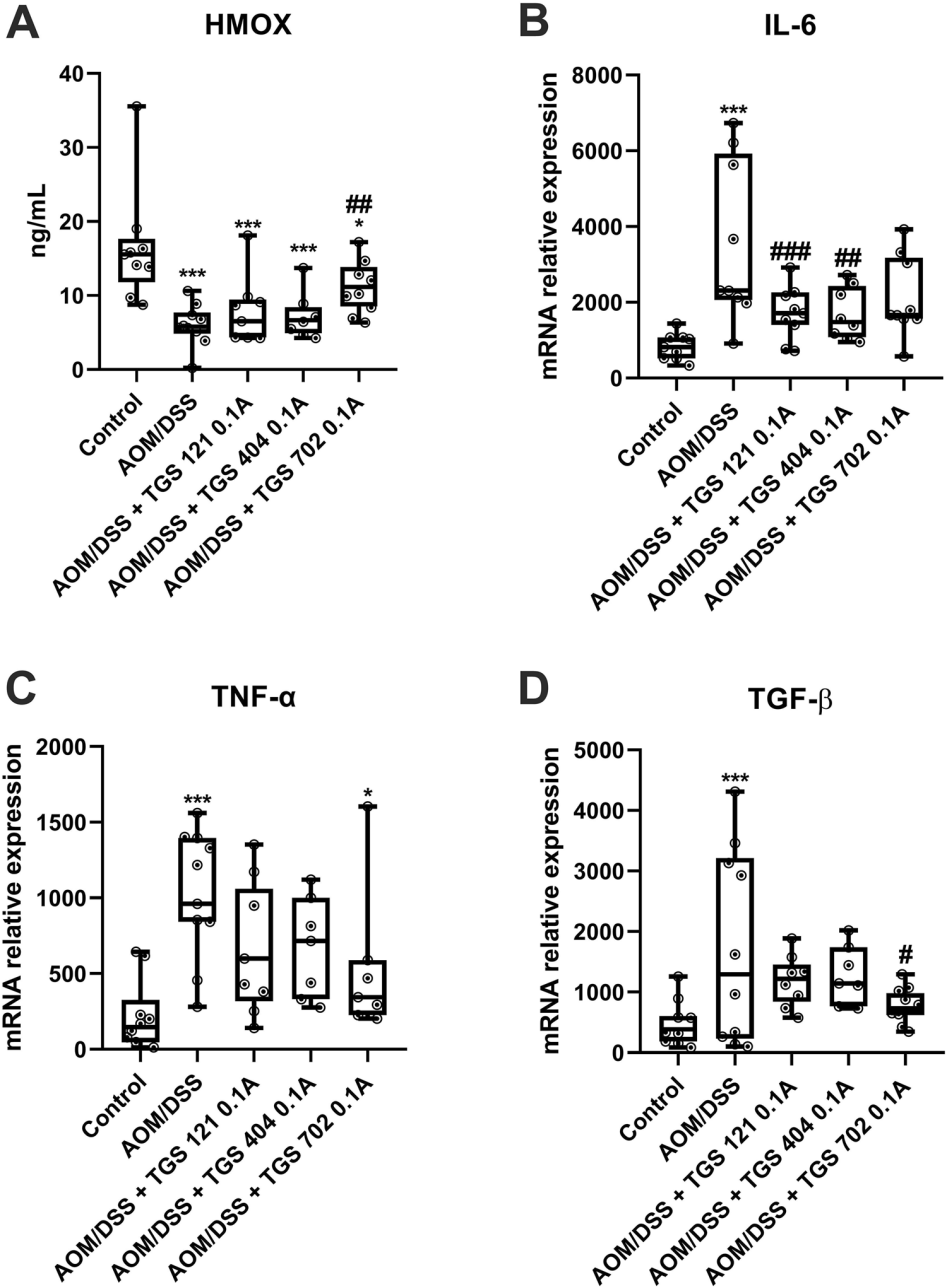
The influence of azoxymethane (AOM)/dextran sodium sulfate (DSS) colitis-associated colorectal cancer (CACRC) induction and TGS complexes treatment on the antioxidant enzyme and pro-inflammatory cytokines expression in the mouse colon: heme oxygenase-1 (**A**), IL-6 (**B**), TNF- α (**C**), TGF-β (**D**) for control, AOM/DSS only mice, and mice with AOM/DSS induced CACRC treated with TGS complexes in dose 1.68 μg/kg (0.1 A). Data in are presented as median with 25th and 75th percentiles with minimum and maximum values (*n* = 6–10). *p < 0.05, and ***p < 0.001 as compared with the control mice; ^#^*p* < 0.05, ^##^*p* < 0.01, ^###^*p* < 0.001 as compared with AOM/DSS only treated animals. Data were analyzed using a one-way ANOVA followed by Tukey’s post hoc test

Then, we evaluated the expression of genes for antioxidant enzyme heme oxygenase-1 and proinflammatory cytokines: IL-6, TNF-α, and TGF-β (Fig. 8). HO-1 level was significantly decreased in each group treated with AOM/DSS and administration of TGS 702 0.1A significantly reversed this effect, as assessed by one-way ANOVA(F_5, 38_ = 12.35; *p* < 0.001) followed by post-hoc Tukey test. AOM/DSS-only mice were characterized by an increase of IL-6 compared to the control group, as assessed by one-way ANOVA(F_5, 38_ = 6.66; *p* < 0.001) followed by post-hoc Tukey test. A decrease in IL-6 expression was observed in every group, which received TGS complexes, however, only TGS 121 0.1A and TGS 404 0.1A reached statistical significance (respectively, *p* < 0.001 and *p* < 0.01). TNF-α mRNA levels were increased in AOM/DSS only and TGS 702 0.1A groups compared to control, as assessed by one-way ANOVA(F_5, 38_ = 6.156; *p* < 0.001) followed by post-hoc Tukey test. We observed a trend in lowering the TNF-α levels after administration of TGS 121 0.1A and TGS 404 0.1A compared to the AOM/DSS-only group, however the statistical significance was not reached. TGF-β mRNA level was almost 4-times higher in the AOM/DSS only group compared to the control, as assessed by one-way ANOVA(F_5, 38_ = 4.905; *p* < 0.001) followed by post-hoc Tukey test. Administration of TGS 702 0.1A significantly reversed the effect of AOM/DSS (*p* < 0.05 compared to AOM/DSS only group).

## Discussion

Gold, used as a therapeutic, had high medical significance in Chinese and Middle Eastern medicine centuries ago [21], but recently scientists “rediscovered” its beneficial properties. Anti-inflammatory effects have been well documented in numerous gold(I) and (III) complexes, also by our group [18, 22]. One of these complexes, TGS 121 was proven to exert its anti-cancer functions in an in vitro study on RAS-mutated CRC cell model [23]. We have revealed that TGS121 selectively induced paraptotic cell death by reducing proteasome activity, causing endoplasmic reticulum stress, aggresome formation, and shift from ERK to AKT signaling in the analyzed model. In this study, we proceeded with the evaluation of the therapeutic potential of gold(III) complexes in vitro and an AOM/DSS-induced mouse model of CACRC. The beneficial effects of TGS complexes were expressed by decreasing the viability of colon cancer cells and presenting potent antitumor action in the CACRC model. This is one of only a few studies on gold derivatives in cancer and opens new possibilities in drug design.

Heme oxygenase enzymes catalyze the degradation of heme into simple compounds: biliverdin, ferritin, and carbon monoxide, and are represented by three functionally active isoenzymes, namely HO-1, HO-2, and HO-3. HO enzymes are involved in multiple physiological processes and also show anti-inflammatory properties [24, 25]. The best-known isoenzyme is HO-1; it has been reported across various tissues and cancers to promote or inhibit tumor progression by multiple mechanisms [26]. In animal models, increased expression and pharmacological activation of HO-1 were associated with a reduction of tumor burden and cellular viability. This HO-1 activity was caused by the induction of apoptosis and modulation of pathways, which are involved in the epithelial-mesenchymal transition [27]. In another study, in 1,2-dimethylhydrazine (DMH)-induced CRC mice, the level of HO-1 increased with tumor progression [28]. The enzyme decreased the viability of cells through induction of cancer cell arrest cycle and apoptosis, which was connected with p53 tumor suppressor protein, and also by modulation of cyclin D1, p21, p27, and PKC and Akt pathways. A study conducted by Gandini et al. showed a correlation between levels of HO-1 in tissues and tumor size, as well as the overall survival time of breast cancer patients [27]. Rushworth et al. reported that overexpression of HO-1 inhibited lipopolysaccharide-induced expression of IL-1β and TNFα in human monocytes [29]. Andres et al. analyzed human CRC samples and the correlation of clinical and histopathological parameters and HO-1 expression [28]. They reported overexpression of the enzyme in tumor tissues and its association with a longer overall survival time. By discussed papers, we noted that mice treated with TGS 121, 404, and 702 were characterized by overexpression of HO-1, which correlated with anti-tumor action in the mouse model of CACRC.

IL-6 may be detected in saliva, serum, or other biological fluids as well as solid tissues. Its overexpression co-occurs with almost all types of tumors and is related to tumor growth and malignancy [30]. According to Simone et al., production of IL-6, IL-17A, IL-21, IL-22, and TNF-α is increased in the mouse model of sporadic CRC and associated with overexpression of STAT3/NF-kB pathways [31]. In line, Heichler et al. reported that the activation of STAT3 through IL-6 and IL-11 stimulates CRC tumor development and is associated with poor prognosis [32]. Inhibition of IL-6 by gold was reported in multiple inflammatory diseases. For example, in rheumatoid arthritis, a monovalent gold compound aurothioglucose inhibited IL-6 and IL-8 induction [33]. In rhabdomyolysis-induced acute kidney injury model, rutin and its gold nanoparticles (Ru-AuNPs) were administrated to the mice; in consequence, expression of IL-6 mRNA in groups which received rutin or Ru-AuNPs was lower than in other groups. Notably, the Ru-AuNPs group was characterized by better results at a lower dose of Ru-AuNPs than the group that received only rutin. According to the authors, the anti-inflammatory effect of Ru-AuNPs appears to be connected with the downregulation of the IL-6 gene [34]. In our study, we have confirmed those observations:- decreased level of IL-6 expression after treatment with TGS complexes was correlated with lower macroscopic score and area of inflammation in the CACRC mouse model.

TNF-α is a pleiotropic cytokine produced mainly by tumor cells and macrophages. It may play both an anti- or pro-inflammatory role in the immune system, as well as it may induce anti- or pro-neoplastic effects in all stages of carcinogenesis. Several lines of evidence show that TNF-α is involved in the transformation of inflammation into cancer [35]; moreover, its overexpression is reported in the CRC tissues and correlates with the CRC stages [36]. Furthermore, TNF-α may be used in the prognosis of CRC [35]. Several studies investigated the effect of gold derivatives on TNF-α in cancer. In the 3-methylcholanthrene induced tumor mouse model, a gold‑manganese oxide nanocomposite induced hypoxia in the tumor microenvironment, and the level of pro-inflammatory cytokines: TNF-α, IL-10, and HIF-1α were decreased [37]. Marmol et al. investigated the influence of an alkynyl gold(I) complex on the colorectal adenocarcinoma cell line [11]. The complex induced necroptosis, which was dependent on TNF-α and NF-κB signaling. Correspondingly, we observed a significantly higher expression of TNF-α in AOM/DSS mice, with a notable trend in decreasing its expression by TGS complexes.

Noteworthy, TNF-α can bind either to TNF-R1 or to TNF-R2. The former activates mainly the extrinsic pathway of apoptosis involving caspase 8 and leading to apoptosis of cancer cells; the latter activates the NF-κB pathway that might protect cancer cells from death. Consequently, the expression of both receptors at the surface of cells and intracellular switching between the pathways might determine the overall response to a given substance. For example, in melanoma MeWo cells, a shift in the NF-κB signaling pathway made cisplatin-resistant MeWo cells less sensitive to proteasome inhibitors [38]. In this context, the anticancer effects of complexes tested in the current study, as well as the lack of difference in the effect between two doses of the same compound can be explained.

It is generally known that there are three ligands of the transforming growth factor-β receptor: TGFβ1, TGFβ2, and TGFβ3. The TGF family is responsible for multiple biological processes, like cell migration, differentiation, and growth. These immunosuppressive cytokines are also crucial in tumor development, being involved in tumor initiation, metastasis, and progression [39]. TGF-β has also been reported as the main inducer of epithelial-to-mesenchymal-transition in CRC [40]. There are several lines of evidence that gold and its derivatives have anti-inflammatory and anticancer effects through TGF-β-dependent signaling. For example, in 5-fluorouracil (5FU)-induced oral mucositis in hamsters, AuNPs improved inflammation parameters, and decreased expression of genes encoding TGF-β, TNF-α, COX-2, IL-1β, NF-κB, and SMAD 2/3 [41]. In a study by Zhao et al., the CRC xenograft mice were treated with AuNPs or normal saline for two weeks, then samples from mice were examined. The group that received AuNPs was characterized by reduced production of tumor stromal collagen I and declined expression of TGF-β1, CTGF, and VEGF [42]. Similarly, in our study, we observed a decrease in the expression of TGF-β after treatment with TGS. This clearly shows that TGF-β belongs to the panel of cytokines responsible for the anti-tumor action of the investigated series of gold(III) complexes in the mouse model of CACRC.

In conclusion, in this study, we report a new class of gold(III) derivatives with anti-tumor potential in *vivo* and in vitro models via an increase in HO and proinflammatory cytokines modulation. We have evidence that the TGS 121, 404, and 702 are attractive therapeutic options for CACRC, and warrant further pre-clinical investigations. Future studies on detailed mechanisms and safety may give a strong rationale for possible clinical translation for these new gold(III) complexes.

### Supplementary Information

Below is the link to the electronic supplementary material.

Supplementary file1 (JPG 1095 KB)

Supplementary file2 (JPG 1361 KB)

## Data Availability

The datasets generated during and/or analyzed during the current study are available from the corresponding author upon request.

## Abbreviations

5FUL: 5-Fluorouracil
AOM: Azoxymethane
AOM/DSS: Azoxymethane-dextran sulphate sodium
AuNPs: Gold nanoparticles
CACRC: Colitis-associated colorectal cancer
CD: Crohn’s disease
CRC: Colorectal cancer
DMSO: Dimethyl sulfoxide
DMH: 1,2-Dimethylhydrazine
DSS: Dextran sulphate sodium
HO: Heme oxygenase
HO-1: Heme oxygenase-1
HTAB: Hexadecyltrimethylammonium bromide
IBD: Inflammatory bowel diseases
IL: Interleukin
LC50: Mean lethal concentration
MPO: Myeloperoxidase
MTT: 3-(4,5-Dimethylthiazol-2-yl)-2,5-diphenyltetrazoliumbromide
NF-κB: Nuclear factor-kappa B
OD: Optical density
RuAuNPs: Rutin and gold nanoparticles
SEM: Standard error of the mean
TGF-β: Transforming growth factor-β
