# Supplementary figures and images for: New gold(III) complexes TGS 121, 404, and 702 show anti-tumor activity in colitis-induced colorectal cancer: an in vitro and in vivo study
Source: Pharmacol Rep. 2023 Dec 11;76(1):127–39. doi: 10.1007/s43440-023-00558-1 (PMC10830623; doi:10.1007/s43440-023-00558-1)

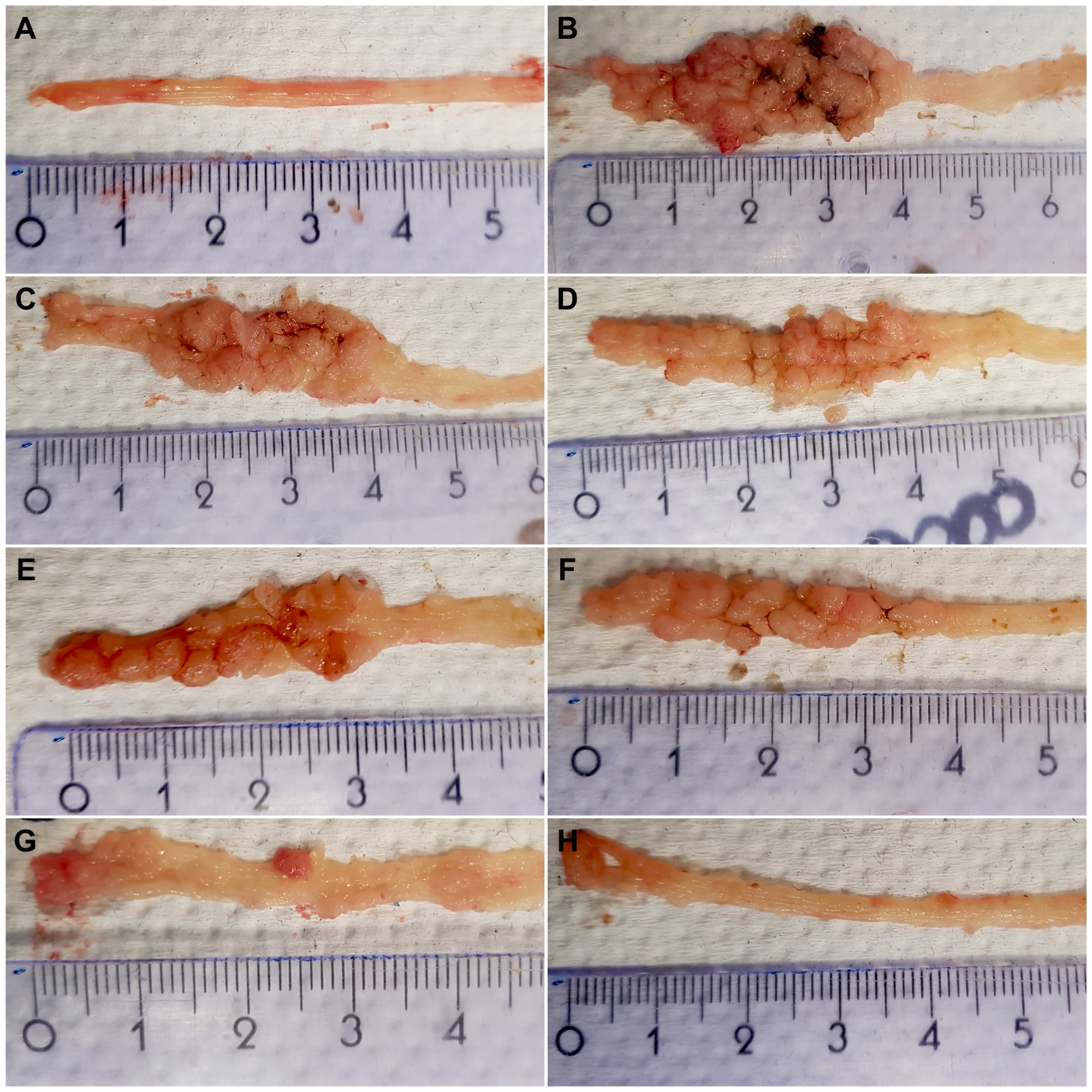

Supplement: Supplementary file 1 — Supplementary file1 (JPG 1095 KB) [file 43440_2023_558_MOESM1_ESM.jpg]

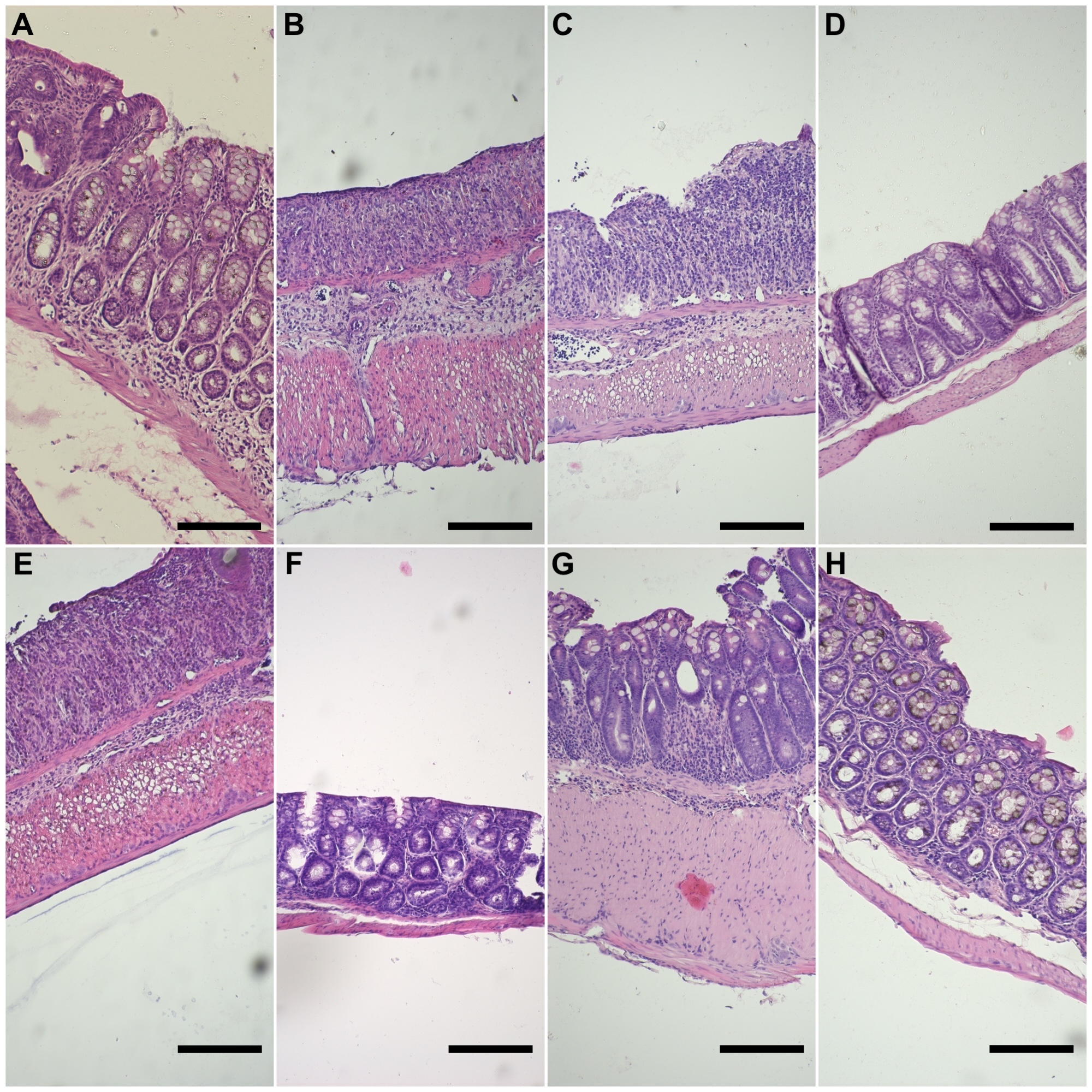

Supplement: Supplementary file 2 — Supplementary file2 (JPG 1361 KB) [file 43440_2023_558_MOESM2_ESM.jpg]
